# Titanium‐Based Static Mixer Electrodes to Improve the Current Density of Slurry Electrodes**

**DOI:** 10.1002/celc.202200928

**Published:** 2023-01-10

**Authors:** Korcan Percin, Jonas Hereijgers, Nicolas Mulandi, Tom Breugelmans, Matthias Wessling

**Affiliations:** ^1^ DWI-Leibniz Institute for Interactive Materials Forckenbeckstr. 50 52074 Aachen Germany; ^2^ Research Group Applied Electrochemistry and Catalysis University of Antwerp Universiteitsplein 1 2610 Wilrijk Belgium; ^3^ RWTH Aachen University Aachener Verfahrenstechnik-Chemical Process Engineering Forckenbeckstr. 51 52074 Aachen Germany

**Keywords:** static mixer, slurry electrode, redox flow battery, electrochemistry, conducting materials

## Abstract

Complex geometries for electrodes are a great challenge in electrochemical applications. Slurry electrodes have been one example, which use complex flow distributors to improve the charge transfer between the current collector and the slurry particles. Here we use titanium‐based flow distributors produced by indirect 3D‐printing to improve further the electron transfer from highly conductive flow distributors to the slurry particles for a vanadium redox flow application. The titanium static mixers are directly coated with graphite to increase the activity for vanadium redox reactions. Increasing layers of graphite have shown an optimum for the positive and negative electrolytes. The application of heat treatment on the electrodes improves the anodic and cathodic current peaks drastically. Testing the highly conductive static mixers in a self‐made redox flow cell results in 110 mA cm^−2^ discharge polarization.

## Graphical abstract

**3D Printed Titanium Flow Distributors**: Highly conductive titanium‐based electrodes are prepared for the vanadium redox flow battery with slurry electrodes. The indirect printing process makes these complex geometries viable. The resulting performance of the battery shows up to 110 mAcm^−2^ polarization current densities.

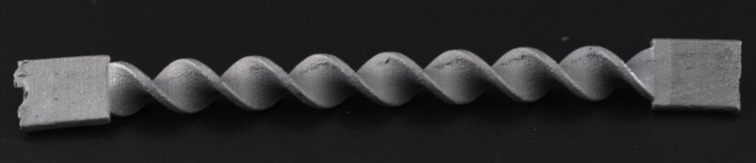

## Introduction

Slurry electrodes, dispersion of conductive particles in liquid electrolyte, have been studied over last century as a replacement of static solid‐porous electrodes in electrochemical applications. One of the earliest applications for slurry electrodes is wastewater treatment.[^1^, ^2^, ^3^] More recently, slurry electrodes can be found in water desalination technologies, such as flow capacitive deionization.[^4^, ^5^] Simultaneously, the interest in slurry electrodes have escalated and broadened to different electrochemical processes, such as energy storage systems. At first, zinc‐air batteries started using slurry electrodes,[^6^, ^7^, ^8^, ^9^] followed by iron redox flow batteries,^[10]^ lithium‐ion technologies,[^11^, ^12^, ^13^] and electrochemical flow capacitors.[^14^, ^15^] One of the latest application for the slurry electrodes has been introduced for a vanadium redox flow battery (VRFB).^[16]^ Yet, there are still many possibilities where the slurry electrodes can be used to replace the traditional electrodes. However, revealing the true potential of slurry electrodes depends highly on understanding the nature of these complex systems. Further studies are therefore essential for the perception of the slurry characteristics and realization in electrochemical systems.

Slurry electrodes are made by dispersing conductive particles in an electrolyte media. The electron transfer is maintained by particle‐current collector and particle‐particle interactions. Continuously appearing and disappearing contacts between the particles and the current collector enable charge transfer to and from the particles, making the slurry system highly dynamic. Commonly, electrochemical reactors utilizing slurries are designed as a fluidized bed system^[17]^ or a continuous flow electrode system.^[18]^ One of the distinct challenges in slurry systems is due to the nature of the dispersion. A slurry dispersion is a shear thinning fluid under laminar flow conditions, resulting in a greatly declined flow velocity in the proximity of the current collectors.^[19]^ The low flow velocity causes the formation of a particle layer on the current collector surface that limits the charge transfer from and to the bulk of the slurry electrodes that are farther away from the current collector surface. This phenomena is also shown by Lohaus et al. with a simulative study.^[20]^ We previously tackled this challenge by using static mixers, an approach from membrane separation processes,[^21^, ^22^] where the static mixers improve the flow conditions by creating secondary flows in the reactor.[^16^, ^23^, ^24^] The static mixers have shown to be a promising approach for the utilization of the slurry electrodes, which was showcased in the electrochemical process of VRFB.

A VRFB is an energy storage process, where vanadium is used as redox active material in both half‐cells. VRFB uses the redox reactions of ${\mathrm{VO}}^{2+}$ –${\mathrm{VO}}_{2}^{+}$ in the positive half‐cell and $V^{3+}$ –$V^{2+}$ in the negative half‐cell. Carbon based paper or felt type electrodes are the typical material of choice in non‐slurry based commercialized VRFBs. In pursue of replacing these standard electrodes with slurry electrodes, we have integrated 3D‐printed static mixers (3DSM) in the flow channels of the battery in a previous work. For an effective implementation of the static mixers (SM), a conductive coating has been introduced. The conductive layer on the SMs allows them to act as current collector as well, extending the possible charge transfer surface from the current collectors to the particles, while simultaneously improving the flow profile by mixing the electrolyte flow. However, this conductive coating showed high electrical resistivity, which may have limited the performance of the SMs.[^16^, ^24^]

Numerous designs for SMs have already been studied,[^22^, ^25^] but the application of conductive static mixers in electrochemical processes is limited, due to constraints building complex structures with highly conductive electrodes, such as metal or carbonaceous materials. Moreover, the electrochemical processes are usually dependent on specific electrode materials, which should show stable behavior under highly acidic or alkaline media and an applied electrical potential. Lolsberg et al. studied the mass transport properties of a stainless steel SM reactor, which is manufactured by selective laser melting. The results of this work indicated an increased limiting current density for a typical redox reaction.^[23]^ Recently, Bayatsarmadi et al. published a novel design for SM, which is designed with the help of computational fluid dynamics. The SM was manufactured from a titanium alloy by electron beam melting method and later coated with platinum for stability. Hereijgers et al. compared different 3D printed nickel SM electrodes against flat and felt electrodes, showing a performance gain over an order of magnitude.^[26]^ These results prove the importance of the static mixer's design on the mass transport properties of an electrochemical cell.^[27]^ Evidently, developments in the additive manufacturing methods are enabling more opportunities in designing electrochemical reactors.[^28^, ^29^]

In this study, we hypothesize that if we use highly conductive static mixers for a slurry‐based VRFB, we can further improve the current density in the system by improving the charge transfer. Therefore, titanium static mixers (TiSM) are manufactured by indirect 3D printing^[30]^ and a specifically designed flow cell is used to evaluate the polarization behavior of the highly conductive SMs for a slurry‐based VRFB.

## Materials and Methods

### Materials

Epoxy impregnated graphite plates (Mueller & Roessner GmbH, Germany) are used as current collectors. A fumapem^®^ F‐14100 (FuMA‐Tech GmbH, Germany) is used as cation exchange membrane. The titanium static mixers are manufactured by indirect 3D printing and spray coated with a conductive lacquer (Graphit 33, CRC Industries Deutschland GmbH, Germany), to render it electrochemically active. A conductive adhesive, Leit‐C (Sigma‐Aldrich Chemie GmbH), is used to decrease the contact resistances between static mixers and current collectors.

VRFB experiments are conducted with commercially available vanadium electrolyte (GFE‐AMG Titanium Alloys & Coatings) consisting of 0.8 M V(III), 0.8 M V(IV) in total 4.5 M sulfate concentration. Slurry electrodes are prepared by dispersing graphite powder (synthetic, 20 μm, 20 m^2^g^−1^, Sigma‐Aldrich Chemie GmbH) into the electrolyte.

### Static mixer manufacturing

**Figure 1:**
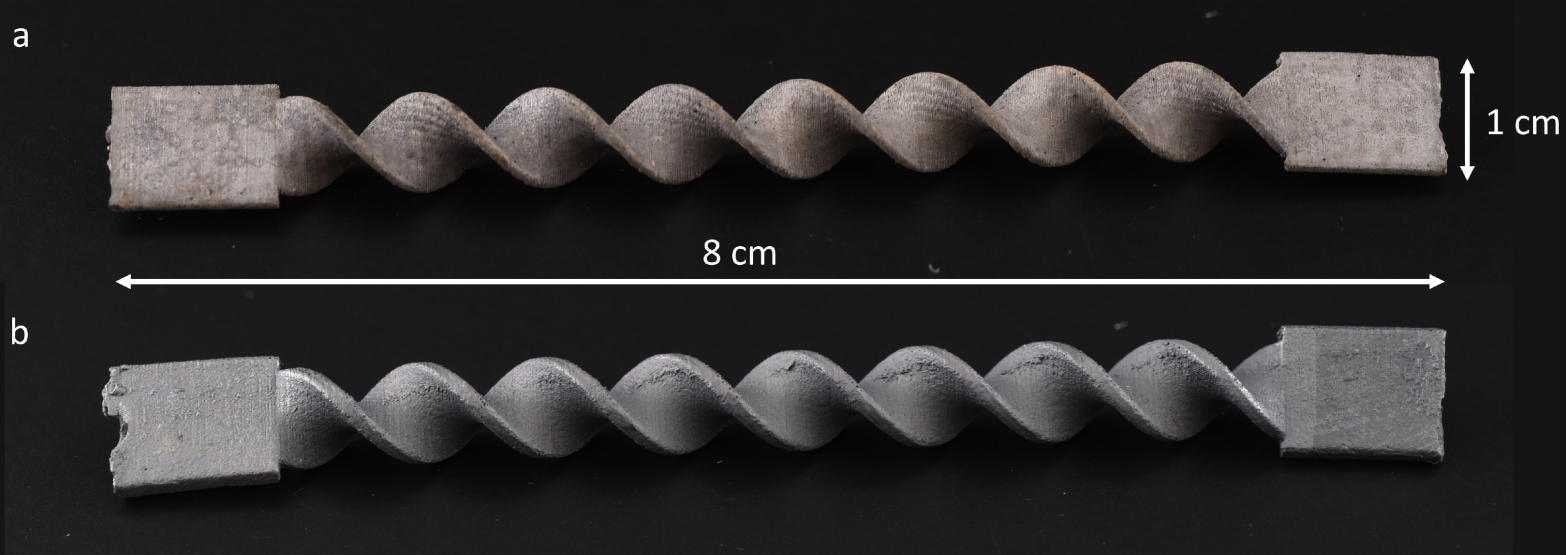
Image of titanium static mixers a) before graphite coating, b) after graphite coating.

The SM are fabricated by 3D printing (Ultimaker) a mold of the SM in Limosolve (Formfutura, The Netherlands), which is subsequently filled with a titanium paste. This paste consists of 79.4 wt.% titanium powder (ASTM Gd2 15 μm, TLS Technik, Germany), 9 wt.% epoxy (Specifix 40, Struers, The Netherlands) and 11.6 wt.% glycerol (Chem‐Lab Analytical, Belgium). After 3 h of curing at 40 °C the mold is dissolved in toluene (Chem‐Lab Analytical, Belgium) and a non‐conductive TiSMs are formed. Finally, a double step treatment is applied to remove the polymer binder and to form conductive bridges between titanium particles. The removal of the polymer binder is firstly achieved at 600 °C for 60 min and secondly the sintering is followed by heating at 1000 °C for 60 min under argon atmosphere. The heating grade is kept constant at 5 °C min^−1^. The TiSM samples are coated with the graphite lacquer by spraying homogenously on each sides. After the application of each layer the samples are dried in a vacuum oven at 30 °C. Manufactured TiSM can be seen from Figure 1.

An identically shaped polymer 3D‐printed SM (3DSM) is used to compare the conductivity and the activity, since previously studied static‐mixers are also 3D printed.[^16^, ^24^] Stratasys Objet Eden 260 V model printer is used with RGD525 polymer. Printed 3DSM samples are later etched in 75 % sulfuric acid for 10 minutes and followed by graphite spray coating. The spray coating is repeated until a reasonable conductivity is reached (15 layers).

### Cell design

**Figure 2:**
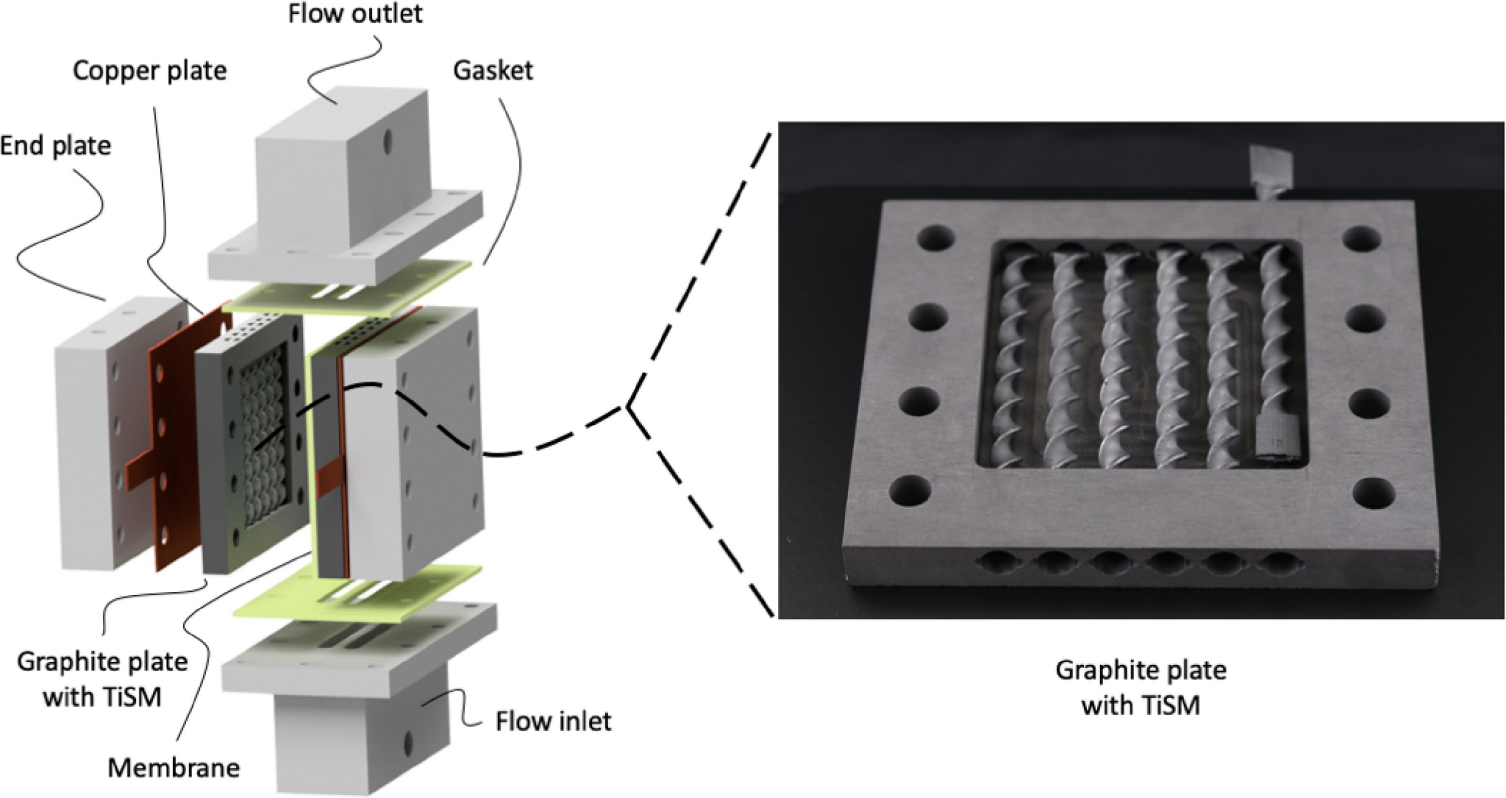
Exploded illustration of the cell (left) with an image of the static mixer inserted graphite plate (right).

The new design consists of two symmetrical half‐cells separated by the cation exchange membrane. Firstly, the current collectors of the module are designed in which a squared flow chamber (55 mm side length) is milled with a 6 mm depth. The depth of the chamber is chosen to enable a practicable manufacturing thickness for the static‐mixers. A half‐cell includes six coated TiSM as flow distributors for slurry electrodes. Insertion of the TiSMs is enabled by having circular holes with vertical dents, which hold the static mixers in place. These holes are also used as the inlet and outlet for the electrolyte that contains slurry electrodes. An exploded assembly illustration of the flow battery can be seen from Figure 2 with an image of the graphite plate. Graphite plate and the titanium static mixers are fixed together with the conductive glue to reduce the contact resistances and to hold the SMs in place.

**Figure 3:**
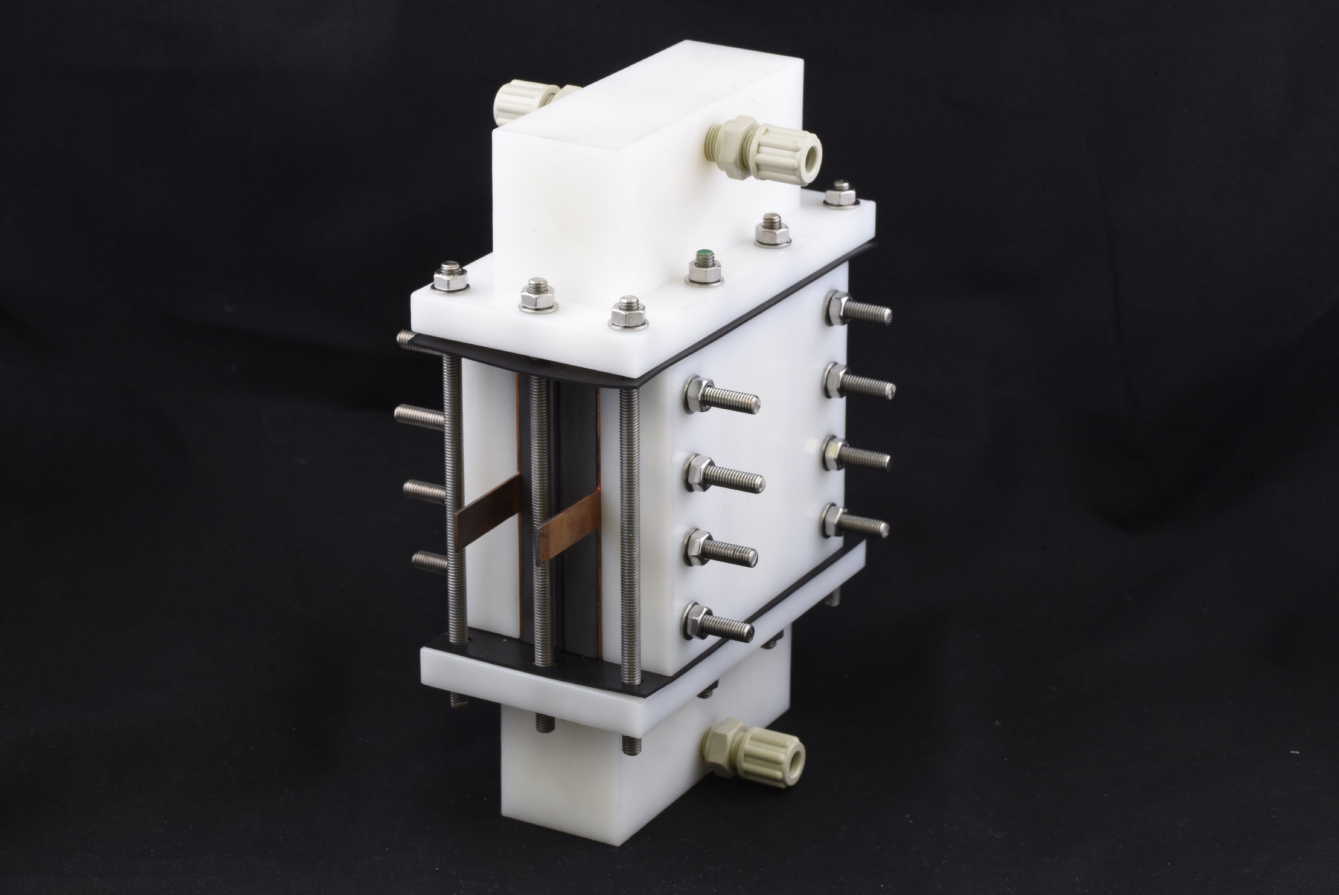
Assembled module for the redox flow battery.

Copper plates are placed at the back of each graphite plate for the connection to the power supplies. The cell is tightened between PVC end plates by means of screws. To ensure an even inflow and outflow, a PVC flow distribution module is designed at top and bottom which delivers a uniform flow for each half‐cell. Gaskets are used to seal between the components. Finally, screws are used to tighten the module. Since, there is two axis compression in the design, the inlet‐outlet chamber has elliptical screw holes, which enables movement of the screws after horizontal assembly is done. The assembled module is presented in Figure 3.

### Surface treatment and morphology

A surface treatment is applied on the graphite coated TiSM electrodes to increase the activity by introducing oxygen radicals on the surface. Therefore, Fenton reaction is used. After the TiSM are spray coated with graphite, the samples are washed in ethanol to remove surface impurities. Then, the samples are dipped in a 0.02 M ferrous solution (pH=3) for 1 h under room conditions. The treatment is followed by addition of 15 mL H_2_O_2_ (30 %) until no bubbles are observed indicating the end of Fenton reaction. Finally, the samples are washed for 30 min with 0.1 M H_2_SO_4_ to remove any attached ferrous compounds and rinsed with deionized water.

The morphology of the static mixers is investigated using a scanning electron microscope (SEM) with an energy dispersive X‐Ray analysis (EDX) detector (Hitachi Tabletop TN3030+). Flat samples for the SEM‐EDX are prepared separately by breaking the samples from the end parts of the SMs.

### Electrochemical characterization

A potentiostat/galvanostat is used with an impedance module (PGSTAT302N, Metrohm GmbH) for all electrochemical measurements. Cyclic voltammetry (CV) measurements are conducted in both negative and positive electrolyte for each layer of graphite coating respectively. The negative electrolyte contains 1.6 M $V^{3+}$ +4 M H_2_SO_4_ and the positive electrolyte contains 1.6 M ${\mathrm{VO}}^{2+}$ +4 M H_2_SO_4_. A typical three‐electrode setup is used with the TiSM or 3DSM as working electrode, a Pt‐IrO_2_ coated titanium felt as counter electrode and a Hg/HgSO_4_ in 0.5 M H_2_SO_4_ electrode as reference electrode. The CV tests are carried out with 0.5 V to 1.8 V vs. NHE in the positive electrolyte and −1.2 V to 0.7 V vs. NHE in the negative electrolyte at scanning rates of 5, 10, 20, 30, 40, 50 mV s^−1^. The stability of the samples are only measured in positive electrolyte (${\mathrm{VO}}^{2+}$ ), where highly oxidative environment may defect the coating on the SMs. Therefore, CV is applied for 50 cycles with 5 mV s^−1^ in a potential range from 0.9 V to 1.4 V vs. NHE.

**Figure 4:**
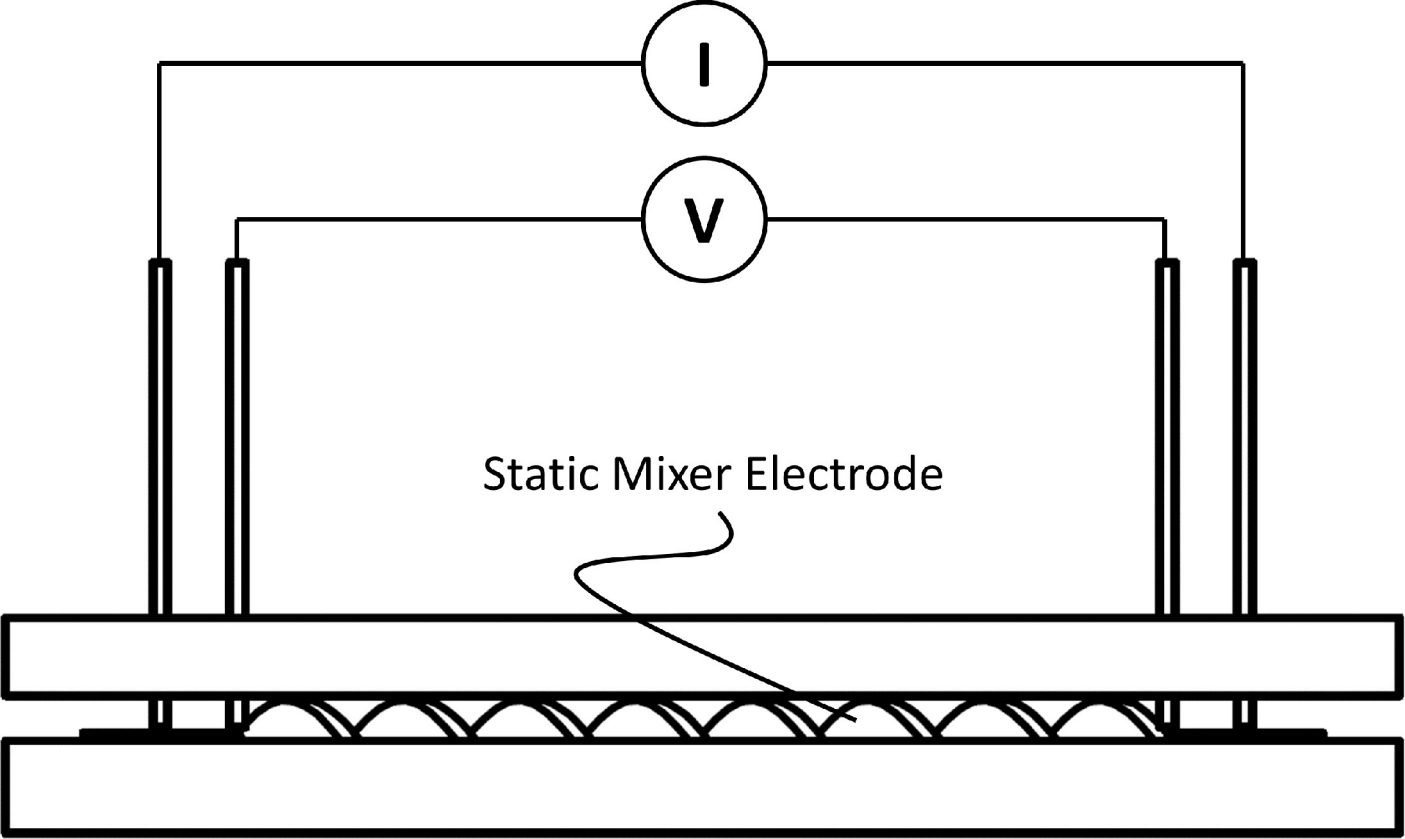
Sketch of self‐made conductivity meter.

Conductivity of the SM electrodes are measured using a 4‐electrode conductivity meter. The conductivity meter includes two electrodes that presses equally on each side of the SMs. A PVC plate is prepared to hold the SMs in place and a lid that is tightened with four screws enables an even pressure on the electrodes. Copper plates are cut and glued in to the top holder. A sketch of the conductivity meter is given in Figure 4.

Polarization tests are conducted in the designed module. Two glass electrolyte vessels are used for each electrolyte sides with a constant N_2_ purge to avoid oxidation of the negative electrolyte. The electrolytes are recycled at a flow rate of 20 mL min^−1^ using a peristaltic pump (Masterflex L/S, Cole Palmer). All the electrochemical tests in the cell are done using 15 wt.% slurry electrodes for each half‐cell. The concentration of 15 wt.% slurry content is chosen according to our previous research, indicating highest current density without viscosity related limitations.[^16^, ^24^]

Electrochemical impedance spectroscopy (EIS) measurements are firstly done in the whole cell with a two‐electrode configuration to calculate the ohmic resistances through the cell. The EIS measurement is done in the frequency region of 100 kHz to 100 Hz at a sine wave signal amplitude of 10 mV before each polarization and charge‐discharge tests. The total ohmic resistance is then used to correct the cell potential values (IR‐corrected potential). Following the EIS measurements, polarization experiments are done with 50 % state‐of‐charge (SoC) electrolytes. A standard VRFB is used with graphite felt electrodes to ensure the SoC. Step‐wise constant current is applied on the cell for 30 s and corresponding cell potential is recorded with a cutoff voltage of 1.8 V for charge and 0.8 V for discharge. The open‐circuit‐potential (OCP) of the cell is set up to the beginning value after each charge and discharge polarization step.

## Results and Discussion

### Surface morphology

**Figure 5:**
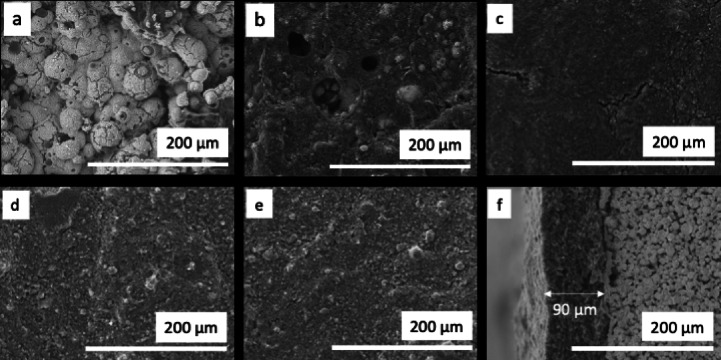
SEM images (500x) of (a) 0 layer, (b) 1 layer, (c) 4 layer, (d) 7 layer, (e) 7 layer treated graphite coating, and (f) cross‐section of 7 layer treated graphite layer titanium static mixers

**Figure 6:**
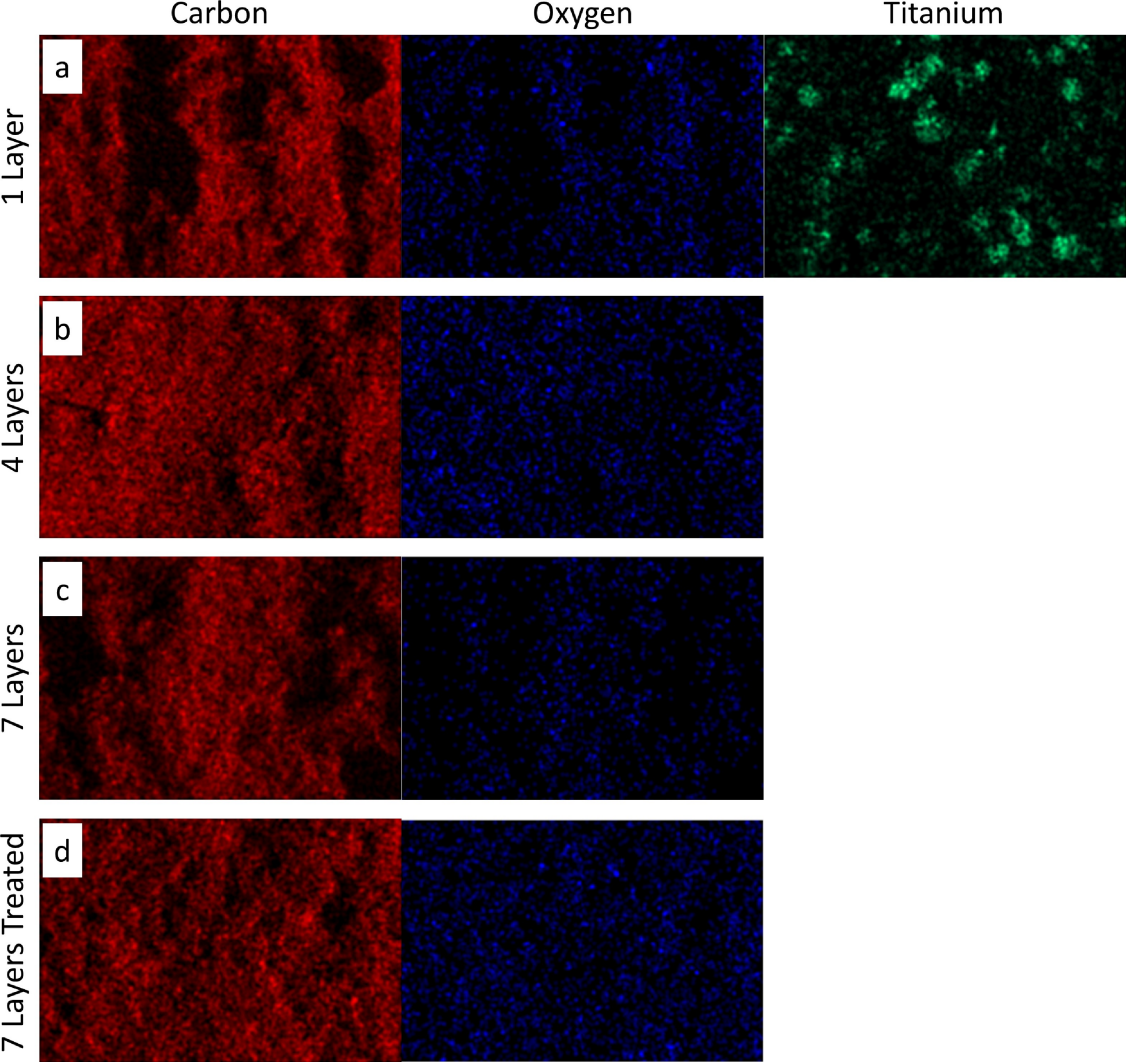
EDX mapping of carbon, oxygen and titanium for (a) 1 layer, (b) 4 layer, (c) 7 layer, and (d) 7 layer treated graphite coating on titanium static mixers.

Figure 5 illustrates the surface morphology of the titanium‐based static mixers with layers of graphite coating before and after surface treatment. The non‐coated TiSM (Figure 5a) clearly shows sinter‐neck formations between the titanium particles, which gives electrical conductivity to the SMs. With the application of the first layer (Figure 5b) most of the surface is already covered with graphite. However, titanium particles are still visible, and the coating consists of considerable gaps. The EDX analysis confirms exposed titanium surfaces in Figure 6a. Smooth coverage of the titanium is achieved firstly at four layers of graphite coating (Figure 5c), where also no exposure of titanium is detected (Figure 6b). However, some crack formations are visible. At the seventh layer, the surface starts to develop a porous structure because of the increased loading of graphite. As it is seen in Figure 5d no cracks are detected on the surface. However, EDX images of the seven layer coating shows some dark spots in Figure 6c. These dark spots are due to uneven surface formation which the EDX system can not detect. Finally, the surface treatment is performed on the seven‐layered TiSM, which resulted in almost no visible surface change, but slightly higher oxygen content on the surface. The surface treatment with H_2_O_2_ is applied to introduce more oxygen‐enriched carbon surface, which provides more active sites towards the vanadium redox reactions.[^31^, ^32^]

The titanium static mixers have a surface area of approx. 15 cm^2^. Seven layers of graphite coating result in 4 mg cm^−^2 graphite loading, which after surface treatment increases to 4.2 mg cm^−2^. Figure 5f presents the cross‐section of the seven‐layer‐coated and treated fiber which has a roughly 90 μm coating thickness. The graphite coating is well penetrated into the titanium SM structure. Upon increasing the number of graphite layers, delamination of the graphite layer is observed after ten layers of graphite coating. Therefore, TiSMs are only coated up to nine layers, of which the electrochemical activity is checked accordingly.

### Electrochemical characterization

After establishing a homogeneous graphite coating on the TiSM, the electrodes are firstly tested for their activity towards the vanadium redox reactions that occur in the battery. The positive and negative electrolytes are tested separately to be able to evaluate the activity of the electrodes for each reaction. The positive electrolyte (catholyte) term is used for the redox reaction of ${\mathrm{VO}}^{2+}$ and ${\mathrm{VO}}_{2}^{+}$ ; the negative electrolyte (anolyte) term is used for the redox reaction of $V^{3+}$ and $V^{2+}$ .

**Figure 7:**
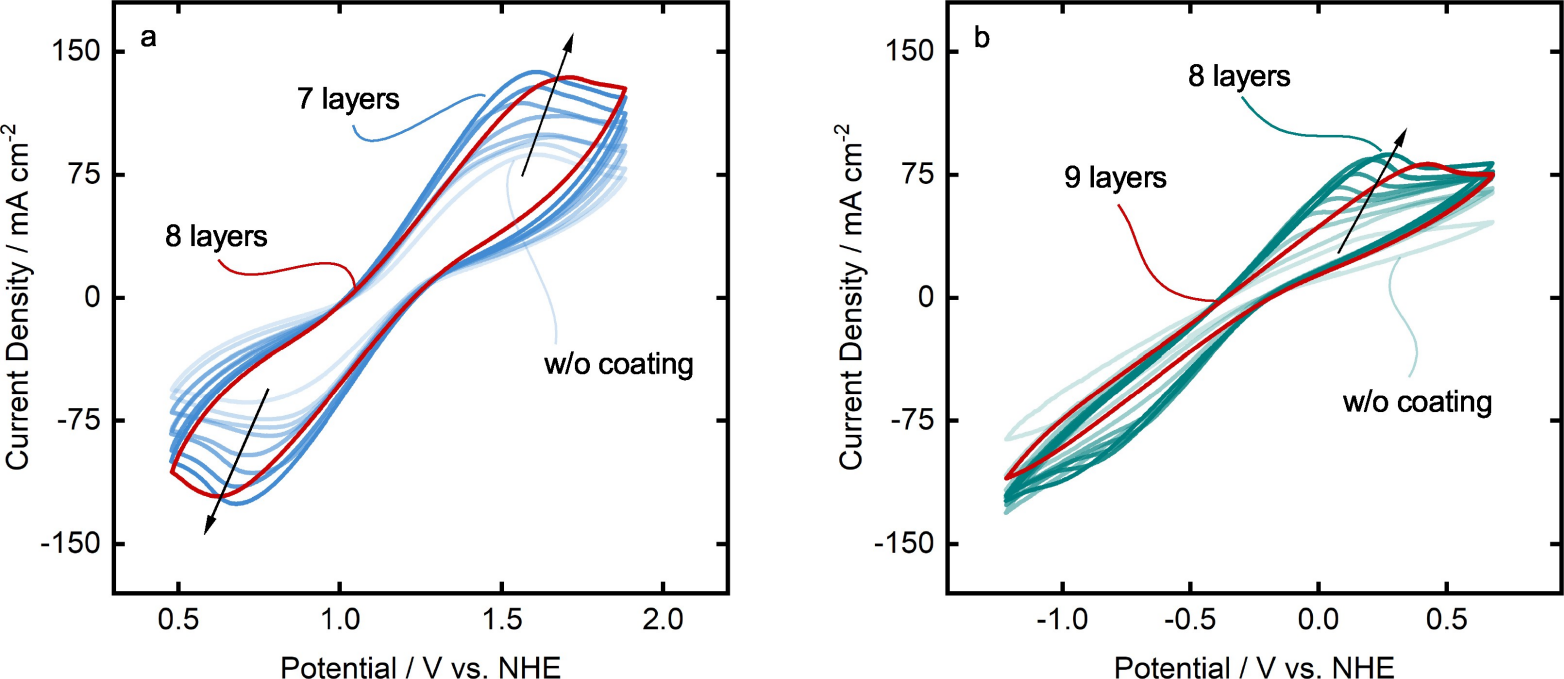
Cyclic voltammetry analysis of graphite coating on TiSMs in (a) positive electrolyte and (b) negative electrolyte at 50 mV s^−1^.

Figure 7 presents cyclic voltammograms of the TiSM with increasing graphite layers for both positive and negative electrolyte. The anodic peak on Figure 7a represents ${\mathrm{VO}}^{2+}$ to ${\mathrm{VO}}_{2}^{+}$ reaction, while the cathodic peak is the reverse, reduction of ${\mathrm{VO}}_{2}^{+}$ to ${\mathrm{VO}}^{2+}$ . Figure 7b shows the anodic peak of $V^{2+}$ to $V^{3+}$ , while the cathodic peak is the reverse reaction. Both anodic and cathodic peaks increase with the addition of new graphite layers. The highest activity is reached at seven layers of coating in the positive electrolyte, while eight coating layers cause the peaks to have slightly lower peak currents (*I_ap_*, *I_cp_*) and higher peak separation (${\Delta E}_{p}$ ). Highest activity for the negative electrolyte is achieved with eighth layers. Nine layers cause the anodic peak potential to increase. During the negative electrolyte tests, hydrogen evolution also accompanies the vanadium reactions and overlaps in the voltammogram. Therefore, it is not possible to clearly detect peaks for the cathodic reaction in the negative electrolyte.

Table 1 summarizes the cyclic voltammetry results of positive and negative electrolyte including peak currents, peak separation and the ratio of anodic and cathodic peak currents. Next to the highest activity, the positive electrolyte also exhibits the lowest $I_{ap}/I_{cp}$ with seven coating layers, which is desired to be as close as possible to be $I_{ap}/I_{cp}=1$ for the electrochemical reversibility of the reactions. The peak separation, however, increases with increasing layers of graphite coating. This increase may be due to the increasing thickness of the graphite coating, which is presumably less conductive than pure TiSM. Similar behavior can be seen for the negative electrolyte. The lowest $I_{cp}/I_{ap}$ value can be observed for the seven layers, even though the highest anodic peak is reached at eight layers. Nevertheless, peak separation shows still an increase with each coating layer. We can so far conclude that seven layers of graphite coating enable electrodes to reach the highest peak current, however with an increasing peak separation potential. In the rest of this work, only seven‐layered graphite coatings on the TiSMs is further investigated.

**Table 1:** Peak evaluation of positive (VO^2+^) and negative (V^3+^) electrolyte voltammograms for the graphite coating.

Layer	Anodic peak		Cathodic peak			
in VO^2+^	I_*ap*_ */mA cm* ^2^	E_*ap*_ */V*	I_*cp*_ */mA cm* ^2^	E_*cp*_ */V*	▵E_*p*_ */V*	I_*ap*_ */I_cp_*
0	86.95	0.91	64.09	0.06	0.85	1.36
3	99.61	0.92	86.52	0.12	0.80	1.15
5	118.70	0.88	106.21	0.07	0.81	1.12
7	136.94	0.93	125.78	0.01	0.92	1.09
8	134.34	1.03	120.51	−0.04	1.07	1.11

Layer	Anodic peak		Cathodic peak			
in V^3+^	I_*ap*_ */mA cm* ^*2*^	E_*ap*_ */V*	I_*cp*_ */mA cm* ^*2*^	E_*cp*_ */V*	▵E_*p*_ */V*	I_*cp*_ */I_ap_*
0	14.89	−0.85	–	–	–	–
3	51.83	−0.70	73.92	−1.36	0.66	1.43
5	66.99	−0.60	83.18	−1.41	0.81	1.24
7	84.36	−0.49	102.28	−1.57	1.08	1.21
8	86.95	−0.41	112.26	−1.74	1.33	1.29
9	80.90	−0.26	–	–		–

**Figure 8:**
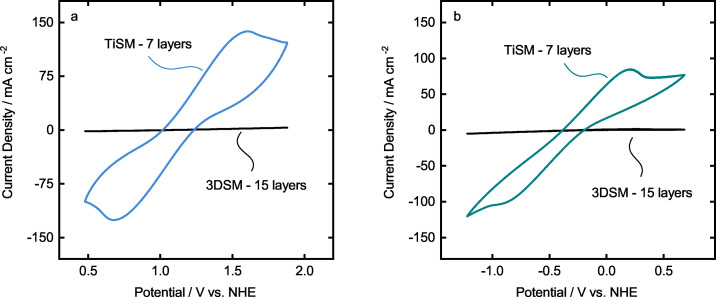
Cyclic voltammetry comparision of TiSM and 3DSM in (a) positive electrolyte and (b) negative electrolyte at 50 mV s^−1^.

Previously, we used 3D‐printed static mixers with a high resistance, which in combination with slurry electrodes still showed improved activity. Here, TiSM electrodes are suggested to be used as highly conductive static mixers. Therefore, we compare the activity of the TiSM and 3DSM to evaluate the differences. Figure 8 presents the comparison of both electrodes in each electrolyte. The 3DSM are coated with 15 layers of graphite to reach a homogeneous coating. Clearly, almost no activity for both positive and negative electrolyte can be detected in comparison to the seven layers coated TiSM electrodes. This result indicates that previously when we used 3DSM and coat them with graphite, the 3DSMs did improve the slurry activity only by changing the flow properties of the cell, not the electrochemical activity. Here we confirm this by the 3DSMs having no comparable activity for both electrolyte reactions.

**Figure 9:**
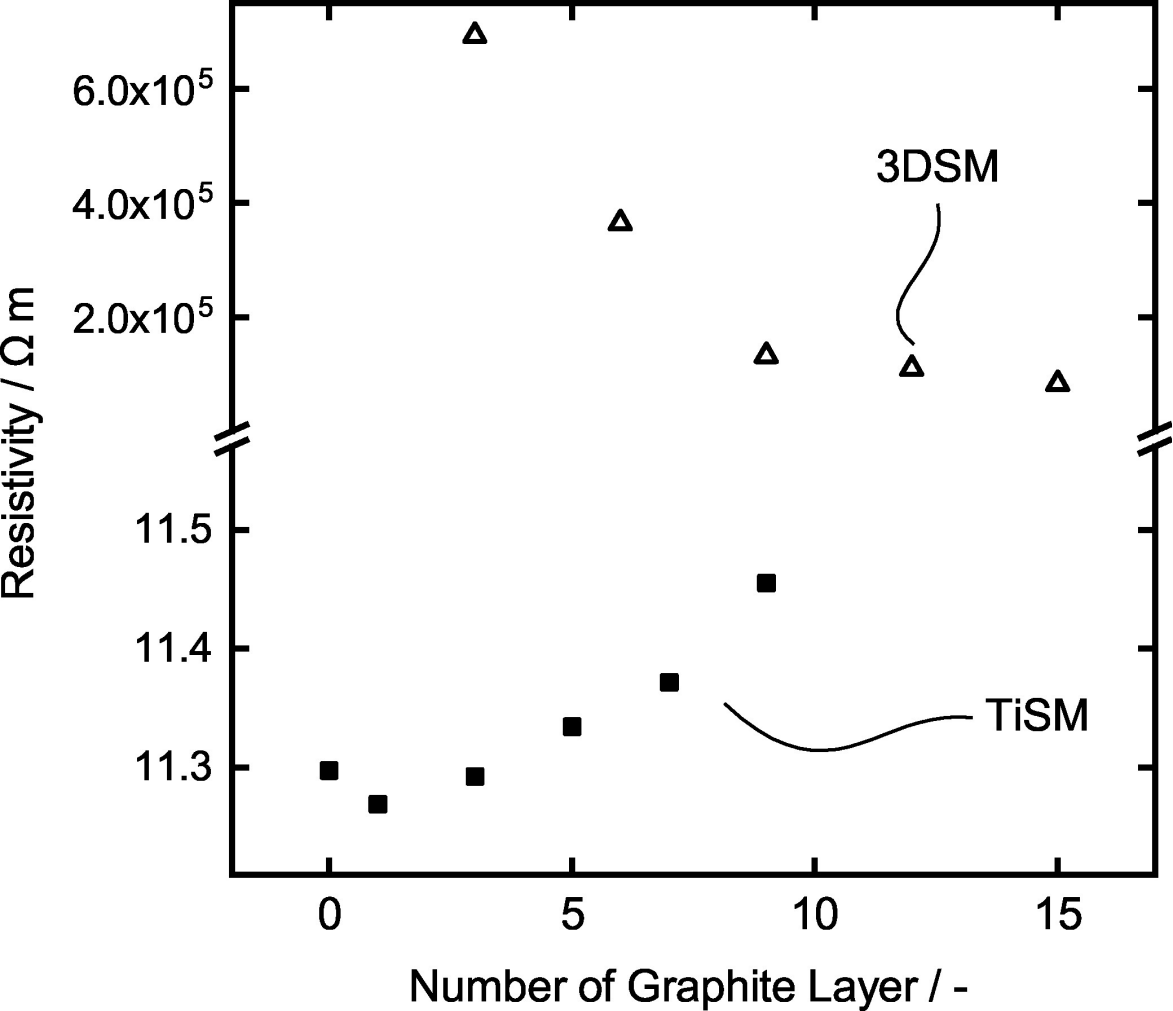
Resistivity measurement of the TiSM and 3DSM with each layer of graphite coating.

To better understand the reason behind the differences between both electrodes, we present the resistivity measurements in Figure 9. The electrodes show a massive difference in resistivity when measured over the whole length of the electrodes. The 3DSM coated with 15 layers of graphite shows about 100 kΩm, while TiSM shows about 11 Ωm. Even though the resistivity of 3DSM decreases significantly after approximately ten layers of graphite coating, it is still considered not to be conductive enough. Consequently, we can assume that the 3DSM samples are not suitable enough to be used as electrodes, thus showing almost no activity towards the vanadium reactions. Interestingly, the resistivity of the TiSM decreases slightly after the first layer of graphite coating, followed by a steady increase in resistivity with the next layers. The decrease in resistivity at the first layer is most likely due to graphite particles filling the porous structure between the titanium particles leading to higher conductivity. However, when the surface is completely coated with the graphite, the resistivity slightly increases since the graphite creates a less conductive surface compared to titanium. The resistivity measurement also confirms the increase peak separation of the TiSM samples, which increases by each layer. Higher resistivity of the TiSM causes higher peak potential separation.

To increase the activity a surface treatment was applied to the seven layer graphite coated TiSMs. It has been previously researched that the graphite surface has a low electrochemical activity which limits the usage for the VRFB. Several different methods have been suggested catalyzing the redox reaction of vanadium by introducing oxygen‐containing groups like C−OH, C=O, and O−C=O on the surface..[^31^, ^32^] These treatment methods include thermal activation,[^33^, ^34^, ^35^] chemical activation,[^36^, ^37^] and modification with metals, CNTs or nitrogen.[^38^, ^39^, ^40^] We choose Fenton's reagent method (${\mathrm{Fe}}^{2+}$ +H_2_O_2_+H^+^) as it is similarly effective, but simple to apply on the electrodes. The samples of TiSMs are prepared and coated with seven layers. Firstly, one sample of TiSM is tested in a CV measurement. After the CV measurement, the surface treatment is applied to the same sample and tested again with a CV measurement.

**Figure 10:**
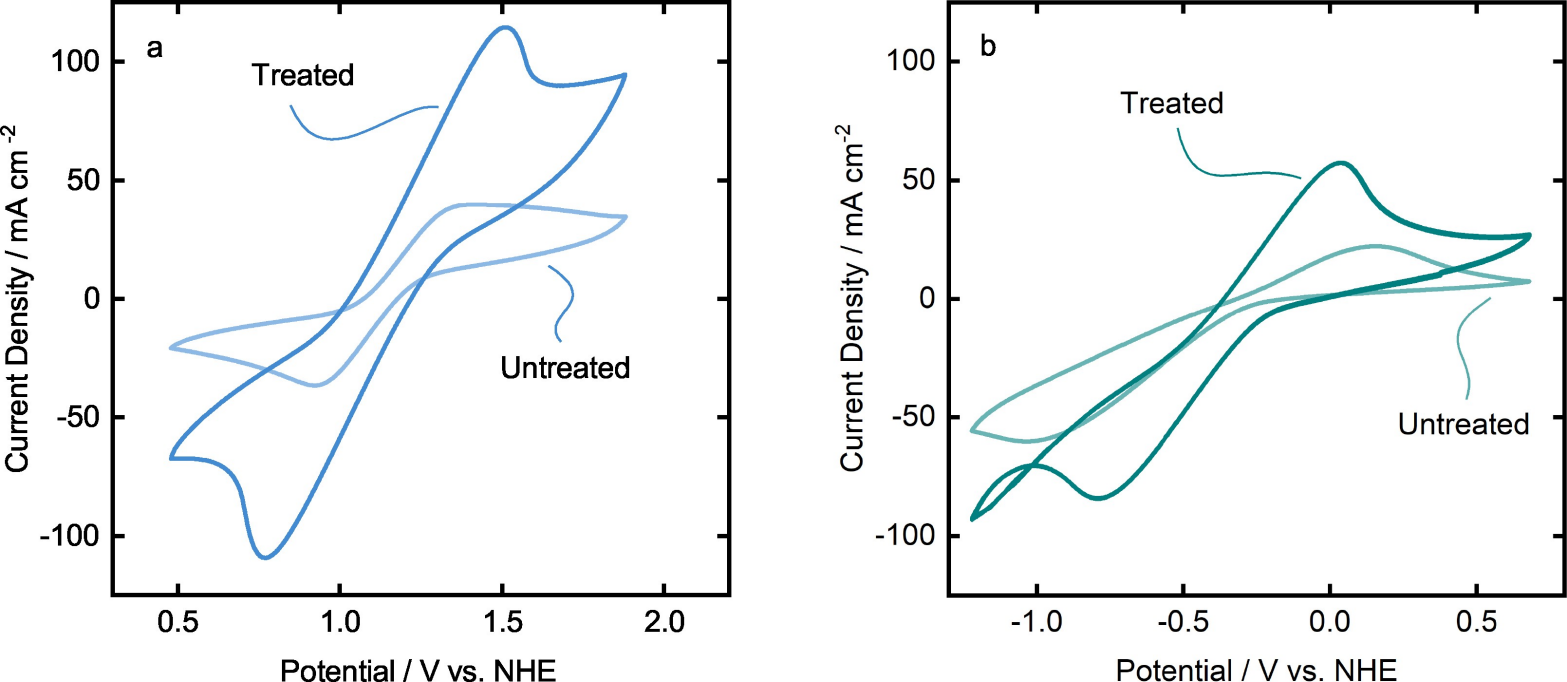
Cyclic voltammetry comparison of treated and untreated TiSMs in (a) positive electrolyte and (b) negative electrolyte at 50 mV s^−1^.

Figure 10 shows the cyclic voltammetry study on the effects of surface treatment on the graphite coated TiSMs. The current density of anodic and cathodic peak at the positive side increases almost with a factor of three after surface treatment. On the other hand, also an increase of current density at the negative side can be observed with almost a factor of two. Table 2 presents the data gathered from the CV measurements to visualize the differences. After surface treatment the peak current density increases to 115 mA cm^−2^, compared to a non‐treated surface where the peak current density was 40 mA cm^−2^ for the positive electrolyte (${\mathrm{VO}}^{2+}$ ). A similar increase can be detected for the cathodic peaks in the positive electrolyte. As seen, the ratio of peak currents also decreases with the surface treatment to 1.05, resulting in a more reversible redox reaction. However, the peak separation ($\Delta E_{p}$ ) increases extremely after the surface treatment, which may be due to the oxidized titanium support becoming slightly more resistive than before. When we investigate the same analysis with the negative electrolyte ($V^{3+}$ ), we observe two‐fold increase in current density for the anodic peak with 57 mA cm^−2^ and 85 mA cm^−2^ for the cathodic peak. A drastic improvement at the peak current ratio and peak separation suggests an apparent positive effect on the treated TiSM samples for the negative electrolyte.

**Table 2:** Peak evaluation of positive (VO^2+^) and negative (V^3+^) electrolyte voltammograms for untreated and treated samples.

Sample	Anodic peak		Cathodic peak			
in VO^2+^	I_*ap*_ */mA cm* ^2^	E_*ap*_ */V*	I_*cp*_ */mA cm* ^2^	E_*cp*_ */V*	▵E_*p*_ */V*	I_*ap*_ */I_cp_*
7 L untreated	39.68	0.68	36.31	0.26	0.42	1.09
7 L treated	114.48	0.84	109.14	0.09	0.75	1.05

Sample	Anodic peak		Cathodic peak			
in V^3+^	I_*ap*_ */mA cm* ^2^	E_*ap*_ */V*	I_*cp*_ */mA cm* ^2^	E_*cp*_ */V*	▵E_*p*_ */V*	I_*cp*_ */I_ap_*
7 L untreated	22.24	−0.53	59.94	−1.68	1.15	2.70
7 L treated	56.98	−0.64	84.42	−1.47	0.83	1.48

**Figure 11:**
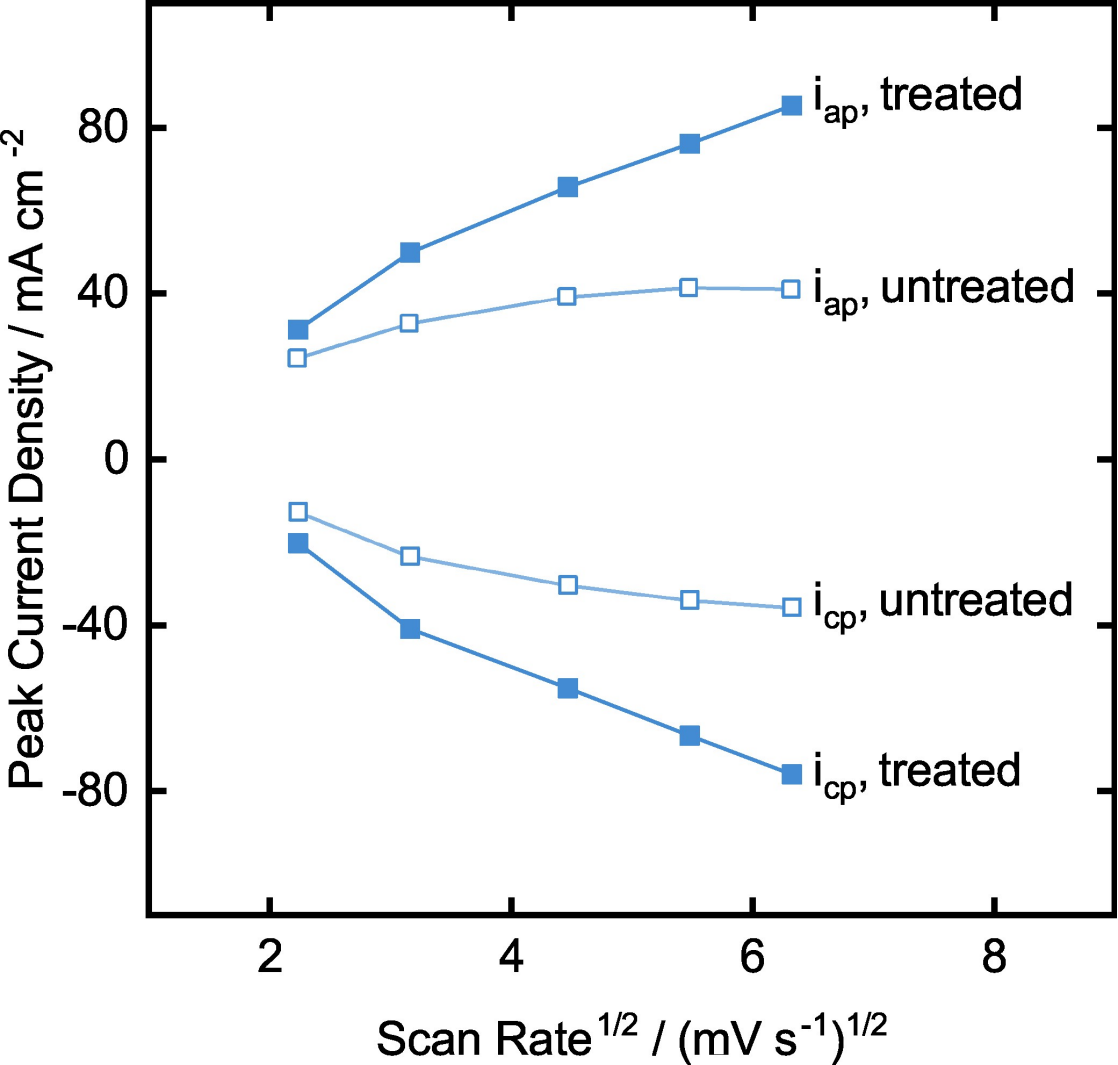
Kinetic analysis of the untreated and treated TiSM samples comparing peak current density versus scan rates.

Subsequently, kinetic analysis of the electrodes is done at different scan rates for the positive electrolyte. Figure 11 shows the dependence of peak current density to the scan rate change. Untreated sample for anodic and cathodic peak current shows a steady increase at the low scan rates and a flat behavior at high scan rates. Such behavior is a clear sign of a combination of mass and charge transport dependent redox reactions. The flattened curve at higher scan rates indicates the charge transport limitation, and a linear increase is a sign of mass transport limitations. After the treatment, both anodic and cathodic peak currents show a relatively more linear behavior, meaning that the positive electrolyte has mostly mass transport limitations occurring at the treated electrodes. This finding is in complete agreement with the previous findings that the treated samples present superior current densities because the treated surface is more active against vanadium redox reactions than the untreated electrodes. Therefore, we can conclude that the surface treatment is a crucial step towards higher current densities. Unfortunately, we could not evaluate the same reaction kinetics for the negative electrolyte, due to poor cathodic peak recognition. However, we expect similar behavior, while the CV measurements of the negative electrolyte and positive electrolyte indicate comparable results.

As the final step of the electrode characterization, we investigate the reproducibility and the stability of the CV behavior of the untreated and treated static mixers (with seven‐layer graphite coating) in the positive electrolyte. This test is done only in the positive electrolyte because ${\mathrm{VO}}_{2}^{+}$ is highly oxidative and may damage the titanium‐based electrodes. Therefore, 50 cycles of cyclic voltammetry with 5 mV s^−1^ is applied on the electrodes and the anodic and cathodic peak currents are inspected. Both samples show stable current densities for cathodic peak currents over 50 cycles. Interestingly, the anodic peak current of untreated samples increases in activity over time, while the treated ones keep constant activity. It implies that the untreated samples reach similar current densities as the treated one after approximately 30 cycles. The reason for this behavior can be explained by electrochemical oxidation of the graphite surface. It has been already reported that the standard graphite electrodes can be oxidized by electrochemical oxidation by applying constant electrical potential in a sulfuric acid electrolyte.^[41]^ The results indicate that under the given conditions, the electrochemical activity can be increased via enriched surface O−C=O functional groups. The same phenomena may be happening since vanadium electrolyte consists of highly concentrated sulfuric acid, and the electrodes are under anodic potential. Nevertheless, this result does not alter the discussion of the stability of the graphite coated and treated TiSM, which shows satisfactory reproducibility.

**Figure 12:**
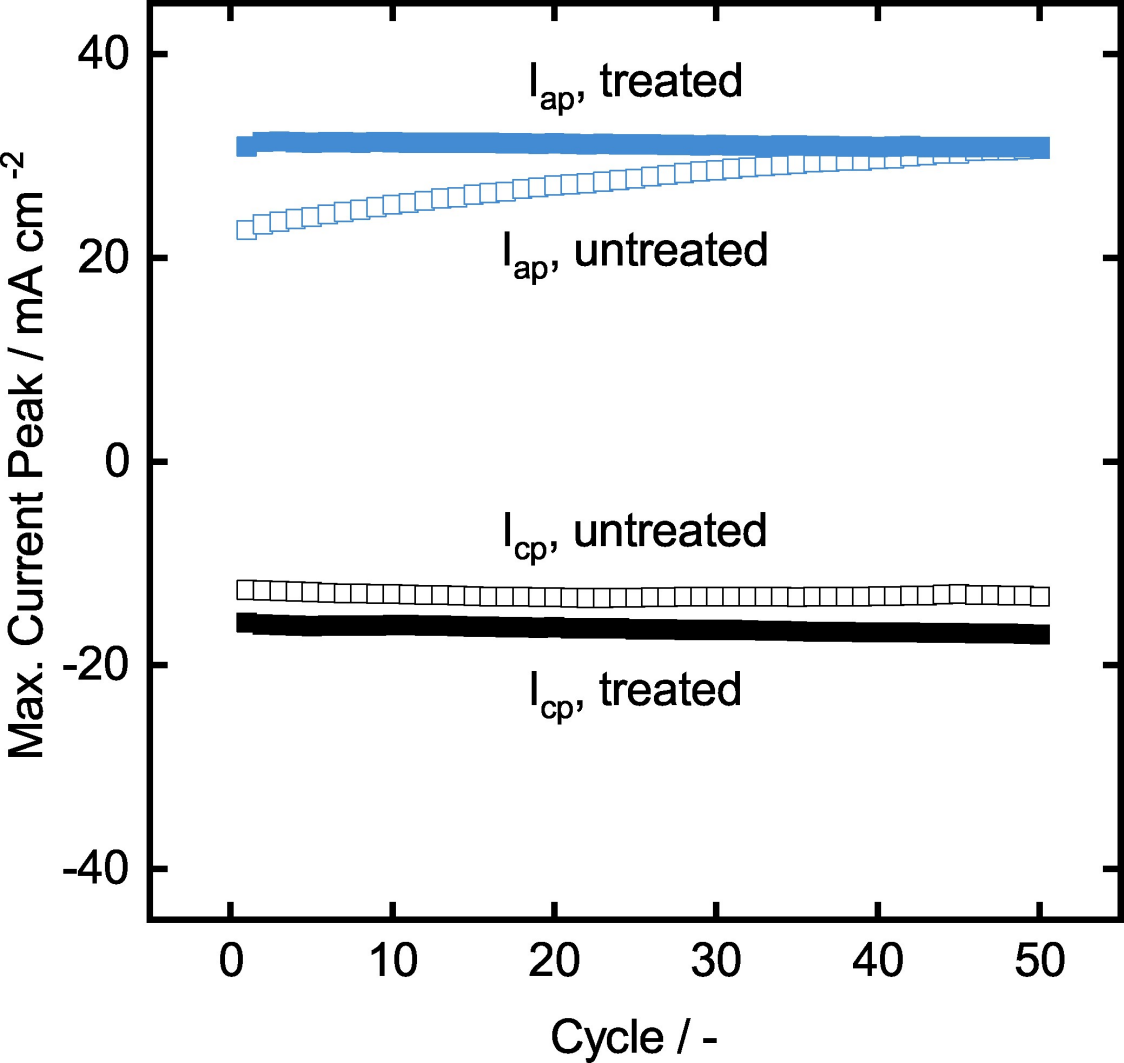
Stability analysis of untreated and treated TiSMs over 50 cycles of CV with 5 mV s^−1^.

**Figure 13:**
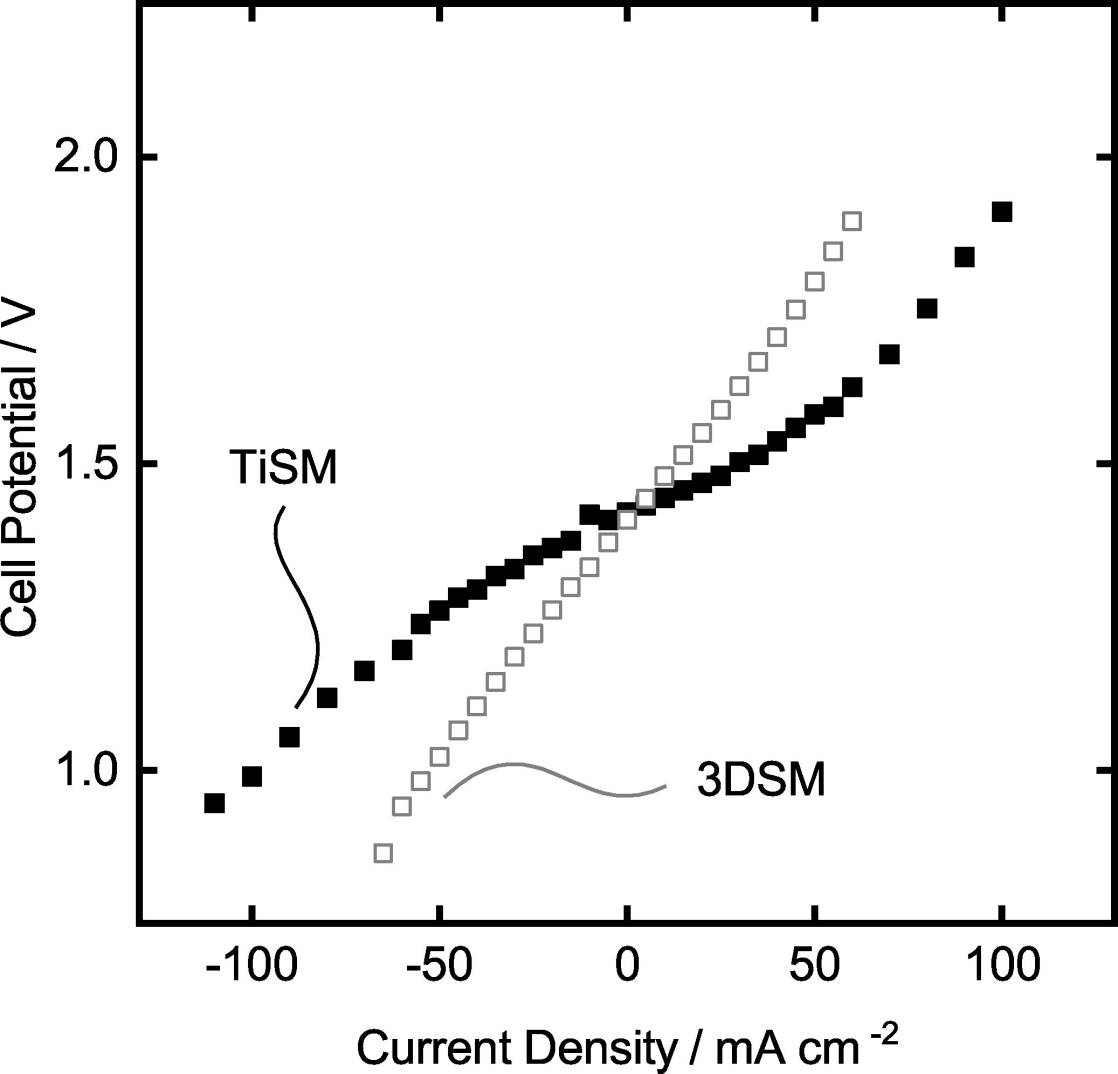
Polarization study of the TiSM electrodes with 15 wt.% slurry electrodes.

Finally, the TiSMs samples are tested for their performance in the newly designed cell, where the slurry electrodes are going to be used as flow electrodes. Therefore, twelve TiSM are coated with seven layers of graphite, and the surface treatment is applied, respectively. The samples were placed in the battery as is seen in Figure 12. The slurry electrodes are prepared by mixing 15 wt.% graphite powder to both the positive and negative electrolyte at 50 % SOC. Before the polarization measurements, an EIS measurement is done to measure the total ohmic resistance in the assembled cell. This value is later used to discard the ohmic losses from the cell potential values. The ohmic losses are the losses coming mostly from contact resistances, electrolyte and membranes. Therefore, a polarization measurement without these resistances gives a better understanding of the electrochemical activity of the cell itself. EIS measurement of the whole cell results in 0.185 Ω resistance, which is relatively high, due to the vast electrolyte chambers. However, since this cell design is the proof of concept for our titanium‐based static mixers, we present the comparison of the TiSM based slurry battery polarization to the 3DSM based slurry battery polarization. Figure 13 presents the evident increase of current density in the TiSM battery. The charge current density shows up to 100 mA cm^−2^, while discharge current density reaches to 110 mA cm^−2^. In comparison to the previously used 3DSM battery, we can improve the slurry electrode activity drastically by using TiSM, because of their higher electrical conductivities. Moreover, the polarization curve of the TiSM indicates a much flatter polarization behavior, which is highly desired for the application in VRFB.

## Conclusions

In this study, we have investigated using highly conductive static mixers ability to improve the slurry electrodes in the vanadium redox flow battery, compared to polymer 3D‐printed static mixers. Therefore, titanium static mixers are produced via mold‐printing method and later coated with a graphite lacquer to have an active surface for the vanadium redox reactions. The graphite coating is later treated with hydrogen peroxide to increase the electrochemical activity. Finally, the TiSMs are placed into a newly designed flow cell to evaluate the polarization behavior of the TiSMs and further they are compared to the previously manufactured 3DSM.

The cyclic voltammetry analysis reveals that we can apply up to seven layers of graphite spray before the delamination and surface deformations occur, which results in a decrease of the activity against both positive and negative electrolyte. When comparing the 3DSM to the TiSMs, we can assume that the 3DSM shows almost no activity in CV analysis, due to the extremely high resistance in the electrodes. Consecutively, the surface treatment on the TiSM electrode results in a three times higher peak current density in the positive electrolyte and a two times higher peak current densities in the negative electrolyte. Finally, the polarization study with the TiSM electrode in the designed cell indicates a superior charge and discharge polarization by reaching a current density to 100 mA cm^−2^ and 110 mA cm^−2^, respectively. The evidence from this study implies the role of a static mixer for using slurry electrodes in a redox flow cell, as a highly conductive SM can improve charge transfer from the current collectors to the particles. In spite of the fact that the design of the new cell shows high resistance due to the electrolyte‐static mixer channels, smaller channel and SM designs for the future may yield even better polarization behavior and respectively charge‐discharge performance can be inspected.

## Conflict of interest

The authors declare no conflict of interest.

1

## Data Availability

The data that support the findings of this study are available from the corresponding author upon reasonable request.
